# Idiopathic high‐flow priapism in a 4‐month‐old infant: A case report and review of literature

**DOI:** 10.1002/ccr3.7985

**Published:** 2023-09-27

**Authors:** Faisal Ahmed, Mohamed Badheeb, Saleh Al‐wageeh, Qasem Alyhari, Abdulfattah Altam, Saif Ghabisha

**Affiliations:** ^1^ Department of Urology, School of Medicine Ibb University of Medical Sciences Ibb Yemen; ^2^ Department of Internal Medicine, Faculty of Medicine Hadhramout University Yemen; ^3^ Department of General Surgery, School of Medicine Ibb University of Medical Sciences Ibb Yemen; ^4^ Department of General Surgery, School of Medicine 21 September University Sana'a Yemen

**Keywords:** case report, high‐flow priapism, idiopathic priapism, infants

## Abstract

**Key Clinical Message:**

High‐flow priapism in pediatric population is rare, yet comprehensive clinical evaluation, along with penile Doppler ultrasound, and cavernosal blood analysis are crucial for accurate diagnosis. Conservative therapy is effective as an initial treatment.

**Abstract:**

High‐flow priapism is considerably rare in the pediatric age group. We report a four‐month‐old infant presented with a prolonged penile erection. Diagnostic confirmation was achieved through the utilization of Doppler ultrasound and cavernous blood gas analysis. We also review published data on the management of this condition.

## Graphical abstract

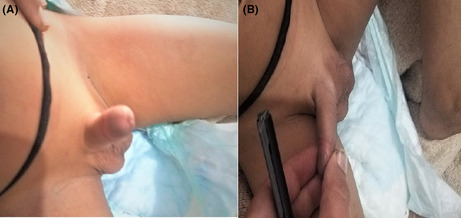

## 1 INTRODUCTION

Priapism is the undesirable, persistent erection of the peins that lasts longer than 4 h. While priapism can often be indicative of an underlying cause such as sickle cell disease, trauma, medication‐induced effects, or metabolic disorders, it can also occur without a specific identifiable etiology in neonates and younger infants.^1^ A comprehensive clinical evaluation, along with penile Doppler ultrasound and blood gas analysis, is necessary to accurately diagnose priapism, allowing for differentiation between low‐flow (LF), also known as ischemic, and high‐flow (HF), or nonischemic, priapism, as well as identifying any underlying causes, if present.^2^ LF priapism is a serious condition; fortunately, its occurrence in infants is exceptionally rare.^2^, ^3^ Despite the relatively low incidence of priapism in the pediatric age group, it is of utmost importance to ensure proper evaluation and management to mitigate potential negative consequences, including sexual impairment in later stages of life.^4^, ^5^ This report describes a case of a 4‐month‐old infant who presented with a prolonged penile erection lasting for 4 days with no identifiable cause.

## 2 CASE REPORT

**FIGURE 1:**
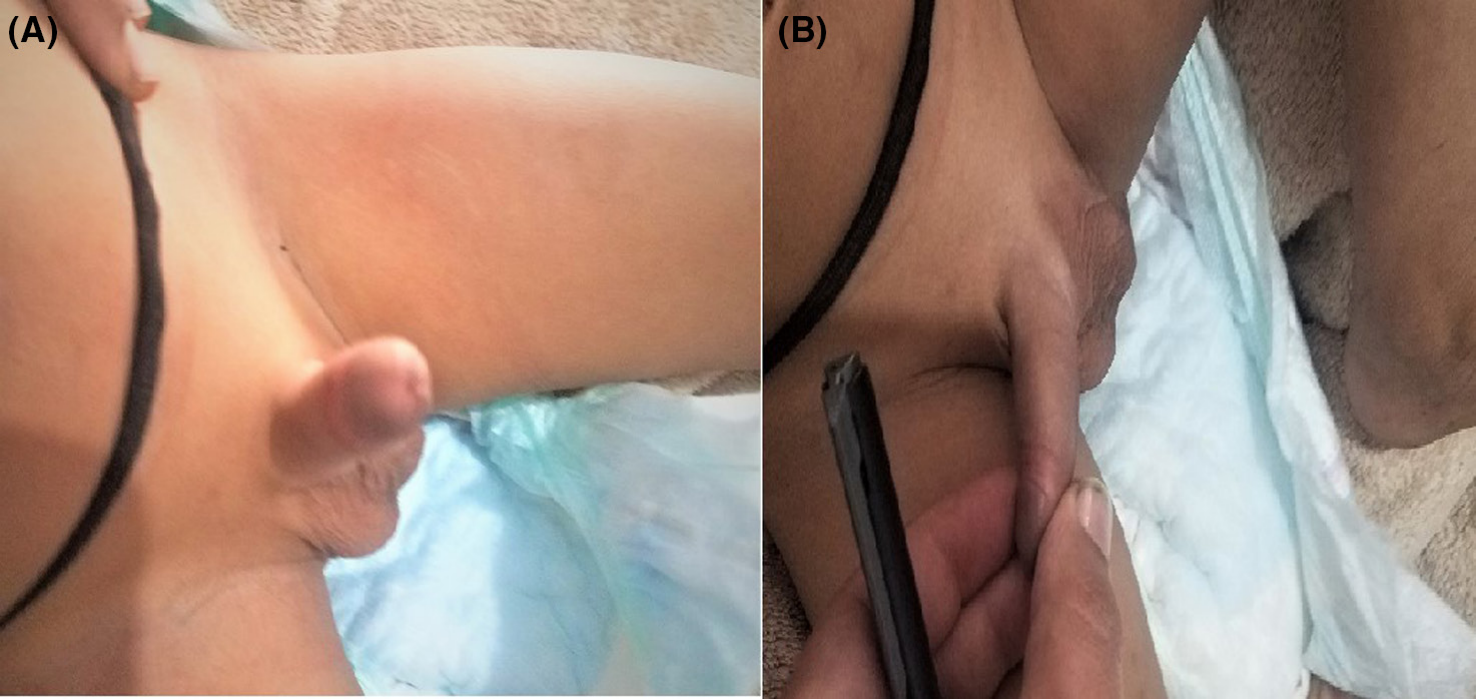
Persistent penile erection n in the 4‐month‐old infant (A: anterior view, B: lateral view).

A four‐month‐old male patient presented with an extended and persistent penile erection for 4 days. As reported by the parents, the patient has had no penile pain, discoloration, or difficulties in urination. Additionally, there was no history of a preceding penile trauma. The patient's medical history included a blood transfusion at 3 months of age, which was done to manage severe malnutrition‐related iron deficiency anemia. Additionally, his sibling had a family history of sickle cell trait. However, there were no previous episodes of priapism reported. During the physical examination, the patient exhibited an erect and non‐tender phallus characterized by a partially rigid corpora cavernosa, a soft glans, and a mild dorsal curvature (Figure 1). Initial vital signs were within normal ranges, and no signs of genital or perineal trauma were evident.

Laboratory investigations revealed a hemoglobin level of 10 gm/dL, a white blood cell count of 6000 cells/mm^3^, a mean corpuscular volume of 77 fL, and a platelet count of 200,000 platelets/mL. In addition, urinalysis and renal function tests were unremarkable. Due to financial constraints, a reticulocyte count and further investigative tests were not performed.

**FIGURE 2:**
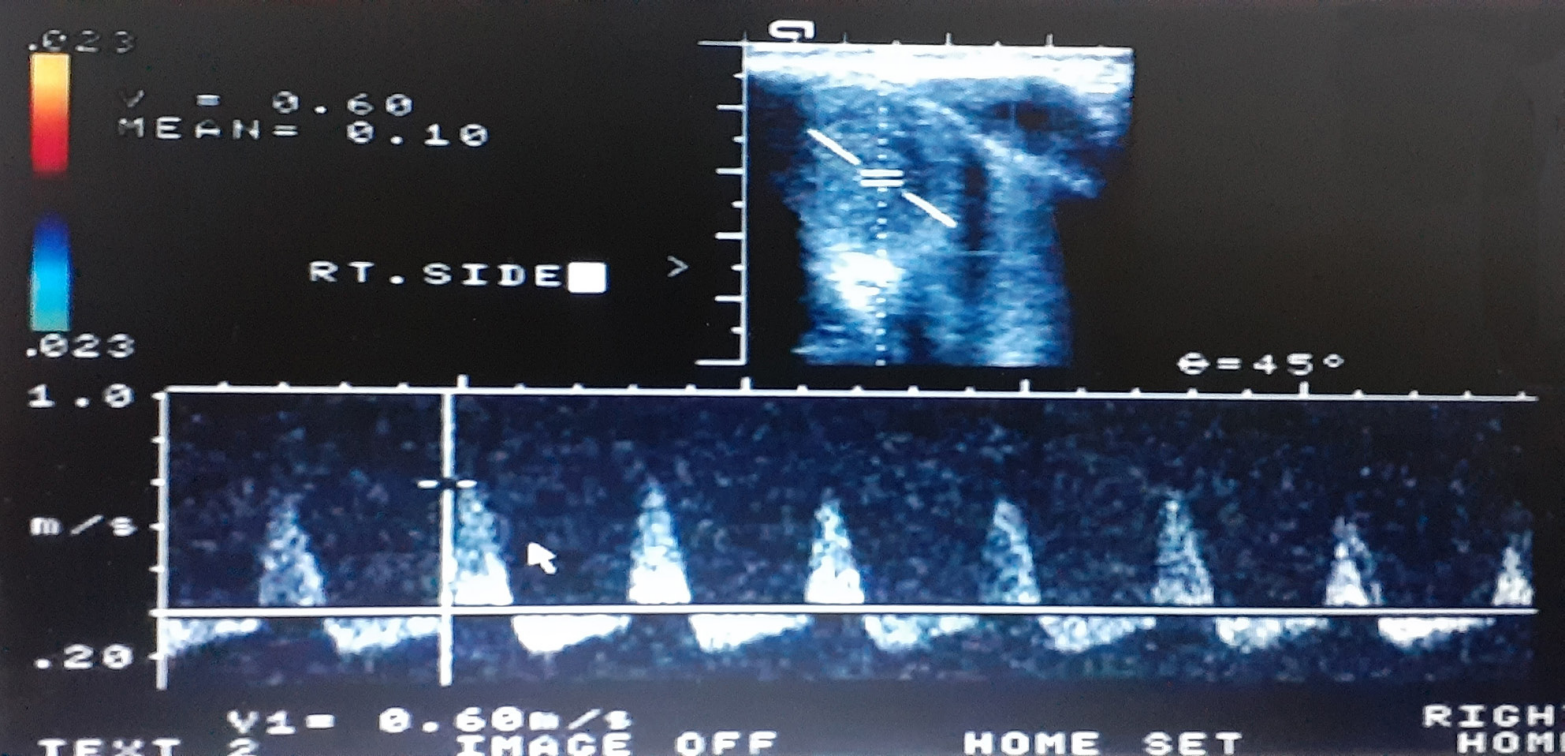
Penile Doppler ultrasound shows a normal arterial flow of the penis in an infant with high‐flow priapism, without fistula appearance.

A Doppler ultrasound of the penis was unremarkable, with no evidence of arterio‐cavernosal fistula (Figure 2). Further, a diagnostic corporal aspiration was performed using a 23‐gauge butterfly needle at the 3 o'clock position, which yielded 10 ccs of bright red blood. The aspirated blood was subsequently subjected to blood gas analysis, revealing a pH of 7.49 (normal range 7.35–7.45), a carbon dioxide (PCO2) level of 32 mmHg (normal range 35–45), and an oxygen (PO2) pressure of 102 mmHg (normal range 88–108). The patient was admitted for further management and was treated conservatively with the application of a penile ice pack and compression. After 2 days, complete detumescence was achieved, and the patient was discharged with an oral antibiotic (cefixime). A follow‐up visit 1 week later demonstrated a normal penile appearance.

## 3 DISCUSSION

Priapism is an unwanted and persistent erection of the penis that extends beyond a duration of 4 h. While relatively uncommon in newborns and infants, it can have detrimental effects on future sexual performance.^4^, ^5^ Common underlying factors contributing to priapism in pediatrics age group include arterio‐cavernosal fistulas, resulting from penile or perineal trauma, blood disorders, like sickle cell disease and leukemia, medication usage, neurological injuries, malignancies, and trauma.^1^ Sickle cell disease is associated with approximately 70% of priapism cases in infants; nevertheless, among neonates and younger infants, the predominance of fetal hemoglobin is protective from the vaso‐occlusive effects of SCD.^6^ Thus, newborns and younger infants should be evaluated for other potential causes including polycythemia, repeated blood transfusions, hypoxia, infections like pyocavernositis, and bladder‐inflammatory pseudotumor arising from catheter insertion.^3^, ^6^

Priapism can be classified into two types based on blood flow: LF (ischemic) and HF (nonischemic). Determining the type of priapism involves a comprehensive assessment of the patient's medical history, including any documented hereditary malignancies, the duration and severity of priapism episodes, presence of pain, previous priapism occurrences, use of precipitating medications, history of genital trauma, or history of blood transfusions.^3^ In our case, the patient has had a history of blood transfusion and a sibling with sickle cell disease. However, the extended interval between the blood transfusion (1 month prior) and the onset of priapism, as well as the patient's young age at presentation (4 months), limited the probability of sickle cell disease to be the culprit.

A comprehensive examination of the patient's genitalia, perineum, and abdomen is critical. In cases of LF priapism, penile engorgement affecting the corpora cavernosa is typically evident. However, corporal infarction, which is of a rare occurrence, may result in engorgement of both the glans penis and corpus spongiosum. In contrast, during instances of arterial priapism, the corpora cavernosa is engorged and fully rigid. Additionally, the application of pressure to the perineum can lead to penile detumescence, commonly referred to as “Pierce's sign.” This sign, which is indicative of arterial priapism, is predominantly observed in children.^3^ In our case, the physical examination revealed a fully erect, non‐tender penis with a partially rigid corpora cavernosa, and a soft glans, indicating a HF priapism.

Cavernosal blood gas analysis serves as a valuable tool for distinguishing between different types of priapism, specifically arterial priapism. Differentiating factors include the macroscopic appearance of blood, typically, dark in LF priapism and bright red in HF priapism, and the results of blood gas analysis. In nonischemic conditions, arterial priapism is characterized by a blood pH above 7.25, a PO2 above 30 mmHg, and a PCO2 below 60 mmHg.^3^ These parameters were assessed in our case, and the cavernosal blood gas analysis indicated HF priapism. Additionally, penile Doppler ultrasound findings play a crucial role in the detection of priapism, in which, low‐resistance waveforms are considered indicative of HF priapism, as observed in our patient.^6^ In Table 1, we summarized the main differences of LF and HF priapism.

**TABLE 1:** Differences between high‐flow and low‐flow priapism.

Variables	Ischemic (low‐flow)	Nonischemic (high‐flow)
Pain	Painful	Painless
Palpation	Sturdy	Elastic
Pulsation	Weak to absent	Pulsatile
pO_2_	Less than 30 mmHg	More than 30 mmHg
pCO_2_	More than 60 mmHg	Less than 60 mmHg
pH	<7.0	>7.0
Arterial inflow	Low	High
Venous outflow	Absent	Open
Viscosity	High	Low

Reports of HF priapism in infancy and young childhood are extremely rare. A case report by Griffin et al.^7^ documented a 4‐month‐old male infant presented with a non‐painful priapism that lasted for 1 day with a spontaneous resolution. Similar to our case, the laboratory evaluation showed normal results, and no additional testing was conducted.^7^

As documented in the literature, the majority of cases of HF priapism in infants are of idiopathic nature and do not necessitate active intervention.^6^, ^8^ In instances where an underlying cause is identified, addressing the root cause can lead to the resolution of priapism. In the management of idiopathic HF priapism in infants, observation is typically sufficient and constitutes the primary approach.^6^ This approach was performed on prior case of idiopathic HF priapism in a 9‐month‐old infant, with normal blood gas analysis results. The patient was treated successfully through conservative management.^3^ Nevertheless, in certain cases, alternative treatments such as phlebotomy or partial exchange transfusion may be considered for patients with polycythemia; in addition, ketamine infusion can be considered in selective cases; however, there are noticeable controversies with its benefit^8^, ^9^, ^10^ Surgical intervention, although available for the treatment of priapism, has not been documented in the literature as a viable approach for infants with idiopathic HF priapism.^11^

The initial management of HF priapism differs significantly from other forms of priapism, and primarily involves clinical monitoring. Conservative measures like the application of an ice pack to the perineum or targeted perineal compression can be considered as part of the treatment strategy. However, aspiration, which serves as a diagnostic tool, is not recommended as a therapeutic option.^6^ The initial therapeutic approach encompasses measures aimed at increasing hydration, alkalinization, and providing pain relief. Shunt procedures are typically reserved as a last resort.^12^ For HF priapism, more aggressive interventions such as aspiration/irrigation, injection of sympathomimetics, and the creation of shunts are generally not required. However, in exceptional cases, where HF priapism becomes extremely severe, leading to excessive blood inflow that exceeds the corpora's drainage capacity and results in penile rigidity, aspiration of the corpora may be considered as a final option.^13^ Another promising modality may include selective arterial embolization, which was used in two cases of HF priapism attributed prior perineal trauma, with penile Doppler ultrasound revealed the presence of an arteriovenous fistula within the corpus cavernosum in two cases; selective arterial embolization was performed, with additional embolization required in one case due to the persistence of an arteriovenous fistula originating from the contralateral cavernosal artery, both cases were successfully managed.^12^

The current report highlights the scarcity of nonischemic priapism occurrences in infants and young children, emphasizing the need for careful evaluation and tailored management approaches based on individual circumstances. Further research and additional cases are required to enhance our understanding of the etiology, diagnosis, and optimal management strategies for this rare condition in pediatric populations.

## 4 CONCLUSION

Idiopathic HF priapism is a rare condition in infants and young children. A thorough history, physical examination, penile Doppler ultrasound, and cavernosal blood analysis can accurately diagnose it. Conservative therapy is the first‐line treatment with a high success rate.

## AUTHOR CONTRIBUTIONS

**Faisal Ahmed:** Conceptualization; data curation; investigation; methodology; writing – original draft; writing – review and editing. **Mohamed Badheeb:** Conceptualization; data curation; methodology; writing – original draft; writing – review and editing. **Saleh Al‐wageeh:** Writing – original draft. **Qasem Alyhari:** Writing – review and editing. **Abdulfattah Altam:** Writing – original draft. **Saif Ghabisha:** Writing – original draft.

## FUNDING INFORMATION

None.

## CONFLICT OF INTEREST STATEMENT

None.

## CONSENT

Written informed consent was obtained from the patient's family to publish this report in accordance with the journal's patient consent policy.

## Data Availability

None.
